# A Concise Review of the Control and Assessment of Magnetic Affinity Particle Assembly for Live Cell Analyses: State of the Art and Challenges

**DOI:** 10.3390/ma18102264

**Published:** 2025-05-13

**Authors:** Sorin David, Daniela A. Tudor, Andreea I. Ftodiev, Camelia Bala, Mihaela Gheorghiu

**Affiliations:** 1 International Centre of Biodynamics, Intrarea Portocalelor Nr. 1B, 060101 Bucharest, Romania; sdavid@biodyn.ro (S.D.); dtudor@biodyn.ro (D.A.T.);; 2 Doctoral School of Biology, University of Bucharest, 91 Splaiul Independentei, 050095 Bucharest, Romania; 3 Doctoral School of Chemistry, University of Bucharest, 4-12 Regina Elisabeta Boulevard, 030018 Bucharest, Romania; camelia.bala@chimie.unibuc.ro; 4 Department of Analytical Chemistry and Physical Chemistry, University of Bucharest, 4-12 Regina Elisabeta Boulevard, 030018 Bucharest, Romania

**Keywords:** magnetic affinity capture, magnetic affinity clustering, magnetically tagged live cells, biosensing, in situ analysis, capture efficiency, antibody orientation, target cell quantitation, magnetophoresis

## Abstract

Magnetic particles have gained prominence in biomedical analyses due to their unique properties, originating from the high surface area-to-volume ratio, ease of functionalization, and their ability to respond to an external magnetic field. Despite its impact in affinity-based biosensing, magnetic particle cluster formation is a largely underrepresented topic at the border of materials sciences, engineering, and biology. This mini-review examines the recent literature demonstrating novel assays based on the assembly of magnetic affinity particles and target live cells, fostering biomedical analyses. It highlights the biosensing opportunities of lab-on-a-chip characterization methods for immunomagnetic clusters and novel approaches for improving affinity capture. It critically discusses the specific means for the on–off control of particle-based immune clusters towards rapid, quantitative tools in live cell detection and analysis of their relevance for biomedical applications involving rare cells in patient samples, such as circulating tumor cells (CTC) and sepsis-related microorganisms. The review aims at encouraging research in magnetic affinity clustering control for biosensing and provides an inter-disciplinary perspective on this high-impact field.

## 1. Introduction

Live cell capture and bioanalysis are fundamental for a plethora of applications ranging from pathogen detection and antimicrobial susceptibility testing (AST) to liquid biopsies and immunology. Accuracy and speed of detection, along with technical and instrumental simplicity, are indispensable for point-of-need (PoN) analytical methods. Magnetic affinity capture (MAC) warrants achieving PoN analyses by combining biorecognition (e.g., immunorecognition in its traditional form), magnetic particles (MPs), and electromagnetic fields for separating, isolating, and concentrating specific targets such as whole cells, cell fragments, and biomarkers from complex biological samples [1]. MAC evolved as one of the prominent techniques for sorting, separating, and isolating cells and microbeads for biomedical applications [2].

Figures of merit of MAC and magnetic affinity separation systems include [3] (a) selectivity, (b) specificity as provided by the biorecognition element, (c) tunability (based on accurate control of magnetic field component of MAC) with field intensity and flux distribution optimized for specific cell types and magnetic tag properties, and (d) integration with microfluidic systems and analytic methods [4] for biosensing. Magnetic control covers a wide spatial domain for cells/analyte capture, as the use of MPs ensures increased surface/volume ratios, reduced diffusion times between analytes and their receptors. MAC can be integrated easily with other analysis methods, thus leading to a decrease in the duration and limit of detection of the assays and high throughputs. MAC is essential in target isolation from complex environments, having non-specific and undesired molecules [4]. As such, analytic systems integrating MAC have not only high sensitivity and specificity, cost-effectiveness, speed, and efficiency, but also minimal sample preparation requirements [5]. MAC is a non-destructive technique, and the target can often be recovered intact after capture if further analysis is required [1]. Indeed, the MAC technique has found various applications in research, clinical applications, medical diagnostics, and environmental monitoring, enabling detection, isolation, and concentration of pathogens, biomarkers, and even circulating tumor cells (CTCs) from various samples [2].

The utilization of magnetic materials in the isolation/preconcentration of various molecules and cells, and their use in sample preparations and various techniques for diagnostic biosensors, is a field of effervescence with substantial progress made on controlling the size, chemical composition, surface chemistry, and affinity modification of MPs.

From a materials point of view, defining MP quality features includes effective functionalization and preparation, stability, and tunable physicochemical properties [6,7,8,9,10]. The high versatility of MAC is based on MP coating with affinity molecules, specific to the target of interest, leading to selectivity, sensitivity, and ease of use in the sensing systems. The most used affinity molecules are antibodies, but one can also use aptamers or lectins [6] and various other compounds (ranging from synthetic peptides to bacteriophages) [11]. Throughout this review, magnetic affinity and immunomagnetic terms will be used interchangeably. MPs coated with biorecognition elements bind specifically to the target, creating immunomagnetic aggregates [12,13]. The characteristics of the immunomagnetic aggregates are sensitive to both MP quality and also to sample composition, and provide interesting detection schemes when coupled with microfluidics [12,13,14,15].

Immunomagnetic aggregates can be evaluated using different techniques, either endpoint such as scanning electron microscopy, or those suitable for monitoring, such as fluorescence microscopy, electrical impedance assay, and flow cytometry [8,10,16,17]. Characterization of immunomagnetic clusters is essential for understanding and controlling the individual MP binding efficiency, stability, and overall performance in complex biological systems, guiding optimization efforts towards minimizing non-specific interactions and enhancing the sensitivity and specificity of assays [18,19]. Additionally, it allows for assessing MP stability and reproducibility, which are essential for clinical and diagnostic use, where consistent performance across batches is required [20].

Given the relation between immunomagnetic aggregate characteristics and the sample composition, in particular the concentration and functionality of target analytes, the evaluation of immunomagnetic aggregate dynamics holds outstanding biosensing potential [21].

In this mini-review, we address the problem of tailored design of magnetic affinity capture for selective biological analyses, whereas we exclude biosensing principles that only employ magnetic particles for concentration, separation, or washing steps. We present different methods for the characterization of immunomagnetic clusters obtained in the immunomagnetic assays. We focus on immunomagnetic clusters formed with bioparticles (pathogens and rare cells) and place particular emphasis on the advantages and disadvantages of immunomagnetic cluster characterization for the phenotypic antimicrobial susceptibility testing (AST) problem. We also highlight practical approaches to improving immunomagnetic capture with the help of affinity compound orientation.

As we bring an interdisciplinary perspective to this effervescent field, this mini-review aims to encourage research in magnetic affinity clustering control for biosensing.

## 2. Magnetic Affinity Clustering Control

MAC is a well-established procedure for concentrating and separating target analytes (cells and proteins) from cell cultures and biological samples for more sensitive and faster [22,23] analyses. In particular, MAC became crucial for separating pathogens (such as bacteria) from clinical samples (e.g., urine) and for microbiological quality control in food samples [9].

The specific cell targeting is achieved through tailored functionalization of MPs, while magnetic separation and immunomagnetic clustering enable subsequent analysis. Most of the time, the term MPs is used to include particles with dimensions that vary from several nanometers to several microns. Copious literature addresses nanoparticles (e.g., gold nanoparticles, quantum dots, magnetic nanoparticles) for identifying and differentiating infectious pathogens and the challenges involved in the clinical translation of these emerging nanotechnology-based diagnostic devices [24]. However, at the nanoscale, the control of aggregation processes is less standardizable and hence of lesser general impact.

Due to better-controlled characteristics, commercial availability, and comparable sizes with the target cells, the use of micron-sized MPs is warranted in integrated diagnostic assays, and they are thus the main focus of this mini-review.

### 2.1. Magnetic Affinity Clusters: Design and Functionality

**Figure 1:**
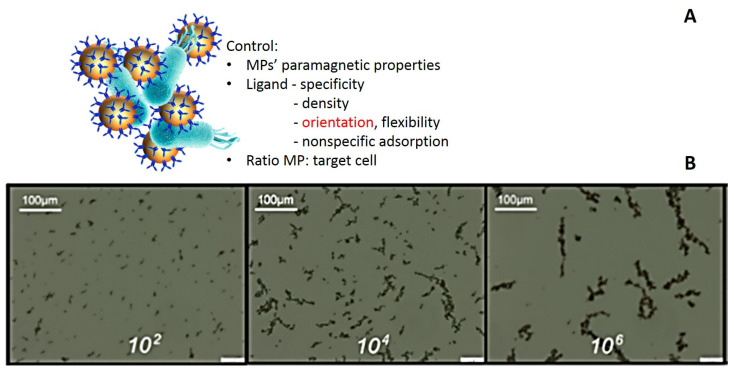
(**A**) Analytical prerequisites for effective affinity clustering: MPs’ paramagnetic properties, ligand specificity, density, orientation, flexibility, nonspecific adsorption, and experimental ratio MP–target cell; cluster morphology and dynamics are dependent on the MP–(bio)particle relative ratios (of both dimensions and concentrations); (**B**) Dependence of cluster characteristics on target cell concentration. Figure adapted from [25], open access Elsevier permission under Creative Commons CC-BY license.

Agglutination processes, based on the ability of antibodies to cross-link antigens and generate insoluble precipitates, have long been used to reveal the presence of specific antigens in biological fluids. Magnetic affinity clustering is a form of agglutination process in which affinity-functionalized MPs bind together as a result of molecules on the surface of the MPs interacting with other (bio)particles [25]. These clusters are typically the result of a process where affinity ligands that decorate magnetic beads are interacting specifically with pair-presenting target (bio)particles, such as cells or complex molecules that possess specific antigens or markers. Once captured, these entities form clusters. Throughout this review, magnetic affinity clusters will refer to aggregates of cells and magnetic particles formed through magnetic affinity capture techniques. The analysis of these clusters can provide important information, such as the efficiency of the capture, the dynamics of the affine interaction, and confirmation of magnetic particle functionalization [26], as well as insights into the viability status of the captured cells [22]. To enable that, careful control must be exerted over the MPs’ (super)paramagnetic properties, ligand specificity, density, orientation, flexibility, nonspecific adsorption, and experimental ratio of MP–target cell (Figure 1), as well as on the analytic setup.

When targeting bacteria with similar sizes to the micron-MPs (approximately 1–2.8 μm), there are few bacteria bound to each MP. In these cases, where the analyte has more than one epitope available for binding to the recognition elements immobilized onto the MP surface, cluster homogeneity can be highly variable. In the absence of the magnetic field, the clusters are string-like, characterized mostly by chains of cross-linked MPs, which occasionally reconnect [25]. The length of these chains depends on the target concentration, according to Figure 1B [25], and even the mere cluster formation is indicative of the presence in the sample of the target cell.

**Figure 2:**
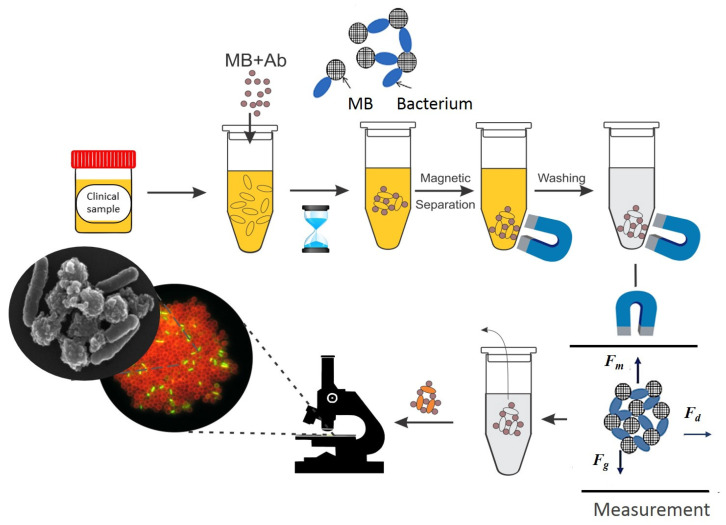
Analytical flow from sample to diagnosis using MAC. The pathogenic cells are extracted from the sample with the help of immunomagnetic capture, and affinity-based aggregates are used in further analysis in either dynamic magnetofluidic sensing platforms or endpoint microscopy analyses. Figure adapted from ref. [27]. Open access.

An integrated analytical flow of immune clusters enables fast quantitation of target cells, expedites end-to-end results, and is suitable for complex samples. For instance, David et al. [27] used functionalized MPs coated with protein G and generic antibodies against gram-specific bacterial constituents to capture, separate, and concentrate the target analytes from the complex clinical sample matrices (i.e., urine samples). The successful capture of *Escherichia coli* (*E. coli*) was demonstrated upon the formation of specific bacterial cell–MP clusters, as presented in Figure 2 [27]. This test yielded, in less than 60 min, information about the bacterial load of the sample tested at concentrations relevant for urinary tract infection analyses. The protocol used was based on fluorescence detection and antibody functionalization of micron MPs. However, the clusters formed by functionalized MPs binding and separating target cells from the solution can be imaged with various endpoint high-resolution assays (with a fluorescence microscope upon staining with a fluorescent dye or with scanning electron microscopy (SEM)) or analyzed in dynamics.

Ideally, the assay should also support optimization efforts. For instance, SEM can also help determine whether the immuno-clusters are homogeneous in size and structure. Uneven cluster formation could indicate issues with the MPs or the antibody coating that must be addressed [28,29].

Given the versatile magnetic control, the analytical flow can accommodate different transitional steps and even homogeneous analyses. As an intermediary step, clusters formed can be integrated in microfluidic dynamic analyses (the measurement step in Figure 2). In such assays, the average cluster size is a marker for the dose response, and individual particle dynamics are cluster size and analyte-dependent. Notably, there are multiphysics models for immunoassay that use microfluidic MP actuation, combined with the principle of agglutination [25]. Accordingly, both the magnetic force (Fm) and the drag force (Fd) in microfluidic setups can be accurately modelled and optimized to improve cluster formation or spatial control [2,30] of MP actuation. Binding of the target directly alters the hydrodynamic volumes of the particles and, depending on the target concentration, leads to aggregation of magnetic particles into clusters. Moreover, cluster morphology and dynamics are dependent on the MP–(bio)particle relative ratios (of both dimensions and concentrations) [25].

The analytical flow is valid also when targeting antigens that are small compared to the MPs (e.g., cell-derived vesicles or molecular aggregates) or much larger than them (e.g., cancer cells). For small analytes, detecting variations of the hydrodynamic properties of MPs following binding of analyte molecules can provide direct sensing strategies [31]. When applying a constant magnetic field, the magnetic moment of individual MPs aligns with the field direction. The magnetic dipolar interaction between particles leads to the formation of particle chains along the field lines. In this way, it is possible to conduct standard sandwich immunoassays on the surface of the magnetic particles and read out analyte concentration-dependent signals directly in the sample solution without requiring washing.

Indeed, magnetic control in microchannels [22] enables microscale sweeping of affinity-functionalized MPs for the efficient capture of rare or low diffusivity targets. Moreover, controlling immunomagnetic aggregates provides effective detection schemes when coupled with microfluidics [12,13,14,15].

Cluster formation was also demonstrated as relevant when designing robust technologies suitable for portability and automated operation [22,32] for fast and sensitive detection of target pathogenic bacteria. The system uses periodic actuation and a lab-on-a-chip platform to induce and record the oscillations of immune-magnetically formed clusters of the target pathogen (e.g., *E. coli* O157:H7). It has a fast analysis time and is amenable to the detection of target cells down to a limit of detection of 10^2^ cells mL^−1^ [32].

External magnetic field control is typically used to modulate particle dynamics, induce acceleration of the recognition rate between grafted ligands and receptors [33], and the bacteria and MP agglutinate through affinity bonding to form relatively large clumps of particles and bacteria. Whether in the washing step (in heterogeneous assays) or in the measurement step (in homogeneous assays), the external fields enhance cluster formation (size and kinetics) in synergy with microfluidics.

The control of the external field supports different sensing approaches broadly covered under the term of optomagnetic biosensors. When stationary fields are deployed, detection can be simply realized by monitoring the extinction of light in the suspension or by evaluating cluster formation via fluorescence.

More advanced optomagnetic biosensors employ alternating magnetic fields to generate periodic movements of magnetic labels, as recently reviewed [34]. Optomagnetic biosensing techniques include magnetic field-enhanced agglutination, rotating magnetic field-based particle rotation, and oscillating magnetic field-induced Brownian relaxation. In these formats, the dynamics of magnetic particles induce an optical modulation that is analyzed by photodetectors, providing information about MPs/clusters, e.g., hydrodynamic size changes of the magnetic particles (carriers or labels).

As such, the control of alternating magnetic fields enables direct detection. For instance, the observed fluorescence intensity of fluorophores bound to the surfaces of the MPs can be modulated by varying the orientation of the particle chains by the applied magnetic field, which changes the relative number of visible to non-visible fluorophores. The biggest disadvantage of these assays is related to the need for labelled analytes or competitive and sandwich (non-competitive) formats. To quantitate the analyte, several alternative methods allow the evaluation of such aggregates. When these particle assemblies are controlled by an applied magnetic field, their signal is modulated, which allows quantification of the concentration and the average size of the magnetic particle clusters without labels. These methods may take advantage of the difference in the diffusion coefficient between aggregated or non-aggregated particles, detect periodic variations of signal under an external field, or rely on a magnetic or impedance [32] detection of aggregates.

These sensing formats will be detailed in the following sections.

### 2.2. Sensing Formats for Magnetic Affinity Clusters

In general, sensing configurations can be grouped by their sensing strategies, i.e., volumetric sensing and surface-based sensing [34].

Among the simplest sensing assays are the ones that rely on stationary fields, nonspecific dyes, and biosensing concepts where optical detection and volumetric sensing involve fluorescent signals. Aside from their simplicity, these methods have as an additional advantage in the fact that they can also support magnetic affinity capture quality assessment during assay development.

#### 2.2.1. Fluorescence Microscopy Assay

Fluorescence microscopy plays a crucial role in characterizing the immunomagnetic capture by providing highly sensitive imaging of the interactions between MBs (coated with antibodies or ligands) and their targets. The primary advantage of fluorescence microscopy in magnetic affinity capture quality assessment is its ability to visualize antibodies binding to the target with high specificity, thanks to fluorescent labels or dyes. It can also be used to evaluate the functionalization of the magnetic beads [27,35,36].

Fluorescence microscopy can provide detailed visual feedback on how effectively the MBs capture and hold onto the target (bio)particles, even under varying conditions, such as different buffer compositions or magnetic field strengths. For visualizing the capture, the bacteria are often labelled with (non)specific fluorescent dyes [27,35,36].

In the study by David et al. [27], the bacteria *E. coli* captured by the magnetic particles were stained with acridine orange (AO), as presented in Figure 2. The obtained clusters were deposited onto a glass slide and observed using a microscope with a B2A fluorescence filter. This approach provides a fast screening for clinical samples since this simple procedure of staining the immunomagnetic clusters with acridine orange can give a response about the status of urine samples, whether positive or negative, for a urinary tract infection. The sample can be considered positive when large aggregates are visible, with bacteria stained green by acridine orange trapped between the magnetic particles, which appear brown-colored [27]. This assay provides information about the bacterial load in less than 60 min compared with at least 24 h for the quantification of the pathogen via the standard urine culture. Importantly, the same study [27] showed that a fluorescence method based on AO staining evaluation with a multimodal reader equipped with a blue fluorescence module (excitation, 460 nm; emission, 515–575 nm) provides information on the capture efficiency and enables optimization of immunomagnetic control assays.

In contrast to time-consuming and imprecise plate count procedures, a rapid fluorescence assay involving AO staining and the assessment of fluorescence of the cells that were not immuno-magnetically separated versus fluorescence of the cells undergoing immunomagnetic separation allows quantitative evaluation of immunomagnetic beads quality towards method optimization.

This work provides an important complement to more classical approaches in which fluorescence microscopy is used to provide information on the successful functionalization of the magnetic beads with an affinity compound and subsequent target capture. In the work by Hasan [36], fluorescence microscopy was used to provide information about the successful functionalization of the magnetic beads with a specific antibody and to confirm the capture efficiency of the target T cell marker (CD3) present in the sample. The CD3 receptors on the T cell surface were stained with green fluorescein isothiocyanate, and the nuclei were stained with 4′ 6-diamidino-2-phenylindole dilactate (DAPI), which is blue. The presence of both colors specific to antibodies and cells enables verification of the capture and specificity of the particular antibodies [36].

In the study by Haghighi et al. [35], magnetic particles coated with Herceptin captured SK-BR-3 cells from peripheral blood that were dyed with carboxyfluorescein succinimidyl ester (CFSE). The authors tested using fluorescence different concentrations of antibodies for immunomagnetic separation to determine the minimum amounts of antibodies for efficient coating. T19 and T25 were magnetic particles conjugated with different amounts of Herceptin: 0.55 mg on T19 particles and 0.009 mg on T25 particles. The T25 had a lower fluorescence observed at the microscope due to a lower antibody concentration than T19 [35].

In an article by Tao et al. [37], magnetosome-like magnetic particles, synthesized via biomimetic mineralization of a protein derived from magnetotactic bacteria and surface functionalized with carboxylated polyethylene glycol, were used to capture the SARS-CoV-2 virus. The virus was stained with a green fluorescent protein, and the particles with red Cy5.5. They confirmed that the particles effectively bind to the virus with fluorescence microscopy since both the green color of the particles and the red color of the virus can be observed in the same cluster [37], sometimes colocalized (yellow color).

The advantages of this method include fast confirmation of less than 1 h (for both the successful functionalization of the magnetic particles and effective target binding) and the minimal sample preparation requirements. The downside of this method of characterization is that is requires labels and the need to couple it with another method, such as mass spectrometry or flow cytometry, because quantitative determination of the number of pathogens is cumbersome and might not be accurate due to the presence of impurities such as dust or chemical contaminants that can emit a fluorescence response even if the target is not present [35,36]. Moreover, fluorescence quantification by clustering is powerful at the nanoscale and, to a lesser extent, at the microscale [35,36].

#### 2.2.2. Flow Cytometry Assays

Flow cytometry plays a significant role in measuring and analyzing immunomagnetic clusters by providing a highly sensitive, quantitative, and rapid method for detecting the presence, size, and properties of immunomagnetic particle aggregates. Flow cytometry allows for the quantification of cluster formation by measuring the intensity of the light scattered (forward and side scatter). The size of the clusters influences the flow cytometry measurements. Cytometers can count individual particles and analyze the relative size distribution of the clusters, providing quantitative data on the concentration of the clusters formed in the sample [21,38,39,40].

Blood samples incubated with magnetic particles functionalized with the antibody anti-CD14 to detect CD14+ monocytes were analyzed using the flow cytometry technique in an article by Leuther et al. [40]. They could quantify the concentration of monocytes using the magnetic flow cytometer technique. This technique is also an essential tool for the characterization of CTCs [41]. In work by Chang et al. [38], CTCs captured by gold particles functionalized by EpCam antibodies were quantified by flow cytometry. This method also helped the authors separate viable CTCs from impurities. They succeeded in capturing 3022 MCF-7 cells and 1100 leukocytes with Fe_3_O_4_@Au-Ab magnetic particles, and 3349 MCF-7 cells and 254 leukocytes with CM-Fe_3_O_4_@Au-Ab. The results of CM-Fe_3_O_4_@Au-Ab were encouraging since cell sorting beads suffer from nonspecific aggregation.

#### 2.2.3. Optical Detection Based on Asynchronous Rotation

Magnetic microparticles self-assemble in the presence of a magnetic field in various structures (including rods and disk-like clusters) and with various dynamics [42,43]. Biosensing concepts where optical detection relies on optical anisotropy changes that are induced by assembly of optically isotropic individual magnetic (nano)particles into chains or clusters, so-called “detection by clustering” optical formats, have been recently reviewed [31,34] and were shown to be equally powerful for both nano- and micro-magnetic beads.

The critical performances of such biosensors are determined mainly by the parameters of biorecognition elements (bioreceptors) and molecular strategies, and are modulated by actuation and sensing principles (MP size and magnetic field strength). Magnetic microparticle-based systems can provide similar limits of detection (LoDs) to those of nanoparticle-based systems, if biorecognition elements with different affinities, i.e., biotin–avidin systems versus antigen–antibody systems (i.e., orders of magnitude different affinities) are used [34].

While not directly related to live cell analysis, the seminal work of Uddin et al. [44] is worth noting. The group of Uddin [44] showed a lab-on-a-disc agglutination assay for protein detection by optomagnetic readout and optical imaging using aptamer-coated magnetic nano- or microbeads. Several elements are significant: the use of both readout and imaging in an aptamer-based agglutination assay used for thrombin detection. The optomagnetic readout of agglutination is based on optical measurement of the dynamics of magnetic nanobeads (MNB) aggregates, whereas the imaging method is based on direct visualization and quantification of the average size of magnetic microbead (MMB) aggregates. Moreover, the aptamers used in this study have a specific affinity to bind to two structurally opposite sites of the thrombin molecule. By enhancing magnetic particle agglutination via application of strong magnetic field pulses, they obtained LoDs of 25 pM with sample-to-answer time (~20 min) and a 10 µL sample volume.

**Figure 3:**
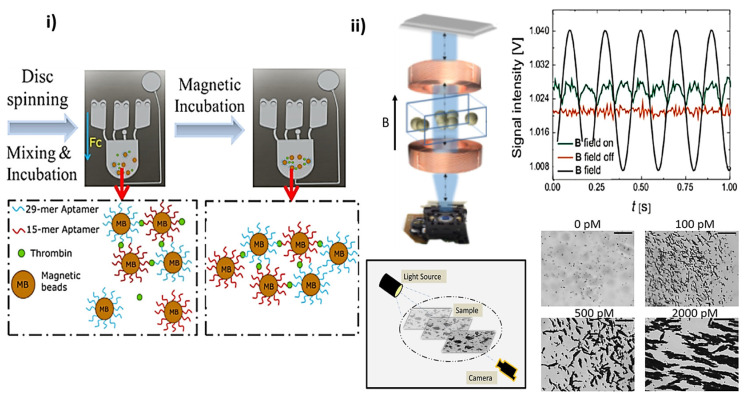
Schematic of the magneto-optic assay for thrombin detection with magnetic actuation affecting light intensity and image details as a function of the target concentration disc. (**i**) Schematic of the functional unit of the optomagnetic readout method for quantifying the nanobead cluster size. (**ii**) Schematic of the functional unit of the optical imaging method for quantifying the microbead cluster size. B indicates the direction of the applied magnetic field. Figure adapted from ref. [44] with permission from Elsevier.

The imaging was based on oCelloScope, an automated microscope with the tilted FluidScope technology (Figure 3ii) that enables scanning the volume of sample suspensions for live cell/particle cluster analysis. It was shown that clusters exhibit asynchronous rotation in a rotating magnetic field, leading to the establishment of asynchronous magnetic bead rotation (AMBR) biosensors.

These AMBR biosensors have been used to detect the growth of individual bacterial cells and their response to antimicrobials. To do so, careful evaluation of the material properties (growth media, supplements, type of reporter MP) was first performed [42]. The stability of the cluster is critical when used as an AMBR sensor, since it influences the rotational frequency that provides the analytic signal. A <1% variation over 2 h was considered relevant, with subsequent requirements to use low amounts of casein hydrolysate (~1%) and rather loosely defined “sticky” beads. When bacteria grow, they alter the drag of the rotating magnetic bead cluster with subsequent shifts in the rotational period (reciprocal of the rotational frequency). This is a key feature of the AMBR sensor, as changes in the drag can be due to changes in viscosity, volume, and/or shape when bacteria attach to the cluster. The rotational period of the cluster changes accordingly when the cluster expands due to increased bacterial concentration or the viscosity of the surrounding fluid changes. Caveats in using this dependence for biosensing are evident since both an increase in the rotational period [42] and a “temporary” decrease [43] were reported for bacterial cell growth.

The high multiplexing potential of the technique is notable, as well as the fact that the use of a rotating field enables “lock-in” analysis of the rotational frequency of self-assembled AMBR biosensors as a function of time. As such, obvious advantages of optomagnetic sensing mechanisms include noise suppression (by performing lock-in detection), reaction acceleration (by magnetic force-enhanced molecular collision), and homogeneous/volumetric detection option. Moreover, optomagnetic sensing can be performed using a low magnetic field (<10 mT) without sophisticated light sources or pickup coils, further enhancing its applicability for point-of-care tests.

A key advantage of magnetic particle labels is given by the possibility to manipulate and actuate the particles by applying tailored magnetic fields, which can be employed to accelerate incubation processes and improve cell capture or enable frequency-selective analysis for improving the signal-to-noise ratio of the measurement signal.

An integrated approach that enables in situ rapid detection, separation, sensitive quantification, and viability assessment of target microorganisms was recently reported [22]. The analytical concept and the related platform are based on a novel integration of magneto-affine selection and electrical impedance assay.

#### 2.2.4. Electrical Impedance Spectroscopy (EIS)

EIS can give important information about the sample’s electrical structure, enabling assessment of clusters of magnetic particles and bacteria [22,32], the bacterial load, and the status of bacterial viability [22,45]. EIS involves measuring the impedance of a biological system, which is positioned between two or more electrodes. This impedance is evaluated across alternating current (AC) frequencies. By analyzing how the impedance changes with varying frequencies, EIS provides detailed insights into the electrical properties of the system, including the resistance and capacitance of biological components. This technique is beneficial for studying interactions at interfaces, such as those between biological samples and sensor surfaces, as well as for monitoring dynamic processes like molecular binding or cell aggregation [22,32,45,46,47]. Specific EIS biosensors function by analyte–bioreceptor interaction, causing a change in capacitance and electron transfer resistance across a working electrode surface. Bioreceptors used in surface functionalization include a wide array of molecules, from antibodies, with or without biotinylation, to bacteriophages, synthetic glycans, and even bio-imprints and bio-mimics of whole cells [48,49], capable of detecting a wide range of analytes from whole bacteria and viruses to cancer cells. A main advantage of impedance biosensors is the unrestricted measurement of the target, with no requirements for the analyte to be enzymatic or specifically tagged to generate formation of electroactive species, as in electrochemical sensing. Disadvantages of impedance biosensors include reproducibility issues, dependence on physical–chemical parameters (e.g., conductivity, temperature), high limits of detection, nonspecific binding [50], and problems with analytic model complexity. Although impedance measurement is straightforward, the complexity depends on the choice of electrode material, base layer construction (for functionalization), bioreceptor conjugation chemistry, type and size of analytes, and complexity of the sample matrix [48].

Selective detection of live pathogens is possible via surface-confined electric field perturbation on surface-engineered impedimetric sensors [49,51] and interdigitated silicon transducers [48]. Slightly outside the focus of this review, these engineered sensors with surface-imprinted polymers (SIPs) [49,51] are worth mentioning since they show potential in direct whole-bacteria detection with large capture ability and relevant LoDs (e.g., 30 cells) in EIS setups. Deposition of SIPs on MPs is, nevertheless, challenging. EIS is a method of characterization in an article by Tsai et al. [45] where magnetic beads with antibodies were employed for the detection of the Dengue virus. They coupled the immunoassay technique with electrochemical measurements of the immunoassay label to quantify the analyte’s concentration based on the electrical impedance measurements. This technique was affordable and facilitated continuous-fluid quantitative analysis. It was tested for four serotypes with the detection limit of 1 ng/mL for serotypes 1 and 4, and 3 ng/mL for serotypes 2 and 3 [45].

The EIS, combined with magneto-immune capture, can also be effective in detecting CTCs since it can differentiate between normal and tumor cells. Based on the dielectric properties of tumoral and healthy cells, EIS can also distinguish between different stages of tumor cells, such as metastasized, invasive, and early stages. In work by Burinaru et al. [41], they tested the capture of CTCs with a limit of detection as low as 3 cells/mL, where anti-EpCAM and anti-CD36 antibodies were used for CTC capture. They evaluated the MCF-7 breast cancer tumor line with three blood samples from dogs with mammary carcinoma. The attachment of CTCs to the magnetic beads was assessed based on the change in impedance [41].

In classical EIS immunosensors, the electrode surface is typically affinity functionalized. Unusual yet powerful approaches are built around formats in which the sensor surface is not set as the working electrode area, as enabled by the use of affinity-functionalized magnetic particles. For instance, antibody-tagged magnetic beads were used to facilitate the migration of target bacteria to an affinity-modified region [52]. The immunosensor was constructed on silanized, nonporous alumina, which separated two compartments with fluid accessibility and was equipped with working and reference electrodes. The antibody-coated magnetic beads with bound bacterial cells were magnetically transported on top of the alumina immunosensor surface to allow for binding and indirect impedance assessment.

This impedimetric method achieved a high binding capability and a low detection limit of 10 CFU mL^−1^. Its complicated setup makes it difficult to translate it into a point-of-care application. However, the setup is valuable since it enables replacement of classical electrochemical formats in novel combinations of filtration with immunomagnetic separation of the bacteria retained in the filters, as demonstrated [28].

**Figure 4:**
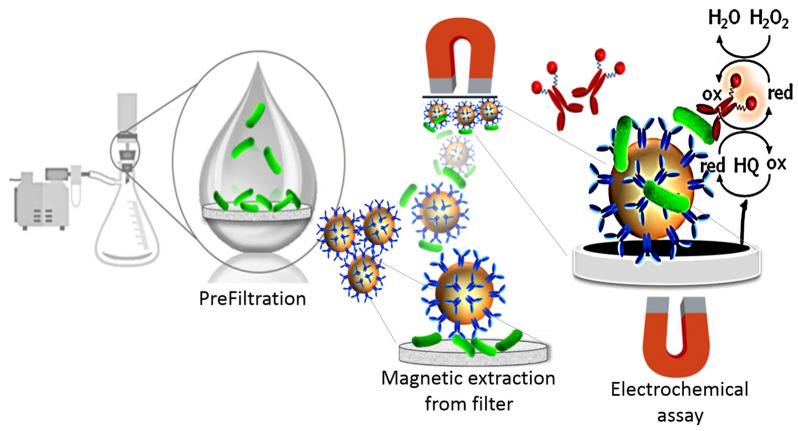
Magneto-electrochemical assay with prefiltration step. Figure adapted from [28] open access under Creative Commons CC-BY license.

Classical electrochemical magneto immunosensing involves calibration and rather lengthy procedures associated with (i) the immunomagnetic separation, (ii) the incubation with the labelled antibody, and (iii) the electrochemical readout [28]. Whereas the protocols may differ in various assays, we exemplify one from a recent paper [28] according to Figure 4. Equal volumes of bacteria-spiked samples (or the filter loaded with bacteria in a prefilter step) and affinity-functionalized MPs, diluted with PBS, were incubated for 1h under gentle rotation. After a washing step, the sample was labelled with Ab-HRP, incubated, washed, and the MPs were resuspended in PBS and the electrochemical readout substrate solution. After 2 min of enzymatic reaction, the solution was placed onto a screen-printed electrode and measured using the square wave voltammetry technique (SWV).

This approach enabled the separation and preconcentration of pathogens from large sample volumes, typically 100–1000 mL, where immunomagnetic separation alone would not be feasible.

**Figure 5:**
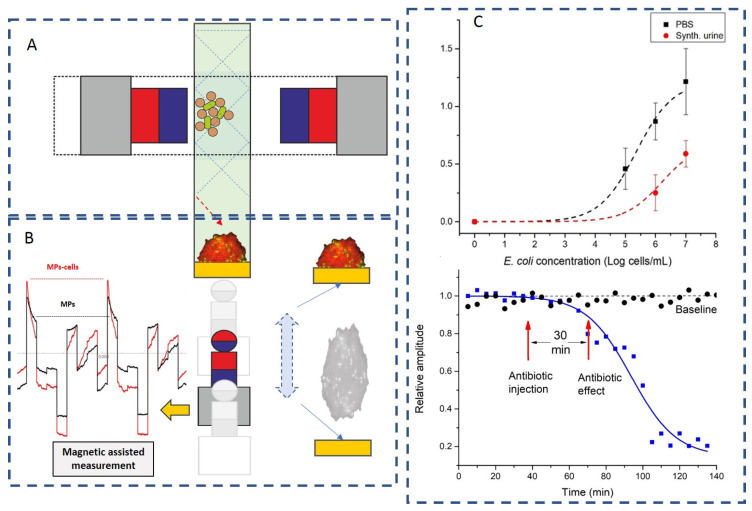
Schematic of the magneto-EIS assay for pathogen detection directly from minimally processed samples (**A**) magnetic actuation for target capture and cluster formation (**B**) magnetic assisted EIS measurement with unfunctionalized electrodes (**C**) target bacteria quantitation and viability assessment; Evolution of the amplitude of the AC impedance oscillations of the magnetically tagged bacteria (*E. coli*) relative to their initial value following exposure to a membrane destabilizing antibiotic—blue. Figure adapted from ref. [22] open access under Creative Commons CC-BY license.

In a radically different approach, David et al. [22] developed a magnetophoretic-assisted EIS concept [32] into an integrated, label-free detection and pathogen viability assessment platform. The setup (Figure 5) involves a chamber with a nonfunctionalized impedance sensor chip where the sample incubation with MPs can also occur, an EIS measurement device, and the magnetic actuation modules for magnetophoresis. These modules can sweep the whole sample to capture and move the MPs for measuring. The formation of clusters is measured in the evaluation region of the chamber where the MAC cluster is oscillated over the EIS sensor.

EIS offers several advantages in characterizing clusters formed during immunomagnetic capture, one of them being label-free detection, which allows for non-destructive monitoring of the clusters without the need for fluorescent, enzymatic, or radioactive markers. This makes it an efficient, cost-effective tool for studying the formation and properties of clusters. As demonstrated in David et al. [22] combination of inline controlled affinity magnetophoresis and magnetophoretically assisted EIS analysis of magnetically tagged microbial cells provides a powerful analytical concept that enables in situ rapid detection, separation, sensitive quantification, and viability assessment of targeted microorganisms from minimally processed clinical samples. The controlled magnetic field actuation determines the periodic and localized coverage of an unmodified working electrode with MP–microbe clusters; thus, the measured electrical impedances relate to cluster size and structure, which depend on the microbial concentration and viability status. The analytical concept and the related platform [22,32] provide high capture efficiency, the ability to provide analytic results within 30 min directly from unprocessed samples (buffer and synthetic urine), and high sensitivity in distinguishing live and dead cells in dynamic exposures. It also eliminates some of the EIS limitations: complex interpretation of impedance data (requiring advanced signal processing and modelling to extract meaningful biological information) and the challenge of non-specific binding or electrode fouling (which can introduce background noise and reduce the sensitivity of the measurements [41,45,46,47]). Fourier analysis and controlled oscillatory patterns eliminate the need for advanced signal processing and modelling to extract meaningful biological information, while the specific chamber design eliminates the challenge of non-specific binding or electrode fouling. Moreover, the combination with microfluidics warrants improved capture efficiencies through enhancement of the encounter rate between target and affinity interface and improvement of binding kinetics [53].

## 3. Biological Analyses Based on Magnetic Affinity Capture

In order to achieve the stringent prerequisites of biological analyses, i.e., low detection limit, target specificity, stability, and reusability, the MP functionalization and related analytical protocols are continuously improved. As demonstrated within various studies published in recent years, MAC is a viable method for bacteria recognition, separation from the samples, and characterization [54,55,56,57].

### 3.1. Bacteria Recognition, Separation from the Samples, and Characterization

In their research, Zeng et al. [58] used immunomagnetic separation and concentration of *S. aureus* in complex matrices characteristic of food samples. Magnetic beads functionalized with immunoglobulin Y derived from hyperimmune egg yolk captured the *S. aureus* bacteria, enabling their collection in PBS. Dodecylsulfate was added to the solution, creating an ATP bioluminescence assay compatible with a portable bioluminescence detector. The limit of detection for *S. aureus* was 3 CFU mL^−1^. This method of separation and concentration had a detection time of under 30 min, confirming the effectiveness of MAC procedures in shortening the time to results, as compared to plate counting, which takes several hours and requires more extended and laborious work [54].

MAC was also used in conjunction with a sandwich fluorescence assay for the separation and analysis of Vibrio parahaemolyticus. In a study by Zhai et al. [59], magnetic particles coated with rabbit polyclonal IgG antibodies (IgG) for *V. parahaemolyticus* in combination with quantum dots coupled with chicken egg yolk antibodies (IgY) against *V. parahaemolyticus* created a sandwich assay for the fluorescence quantification of the bacteria. The quantum dots were used for fluorescence labelling of the bacteria that are captured on immunomagnetic beads. The specificity of this assay was tested using six other bacteria, while the limit of detection for this sandwich assay was 10^2^ CFU/mL [59].

The magnetophoretic-assisted EIS concept developed by David et al. [22] enabled the extraction of calibration curves where the concentration of the bacteria, *E. coli*, immuno-magnetically captured on affinity-functionalized MPs, was directly proportional to normalized impedance data. The same study showed, as proof of the concept, the detection of immuno-magnetically captured fungi *S. cerevisiae* by MPs coated with *S. cerevisiae*-specific antibody down to the concentration of 10^5^ cells/mL. The single-stage detection format, as well as functionalization-free sensors/EIS electrodes, is noteworthy against the fluorescent MP and boronic acid-decorated multivariate metal-organic frameworks [60] or SIP-based *S. cerevisiae* detection [51] formats with lower LoDs (100 and 30 cells, respectively).

As another biomedical application, MAC was successfully used for specific cell removal from various samples. Since the invading pathogens from the clinical samples are usually unknown, enrichment and capture must cover a broad spectrum of strains. For instance, while David et al. [27] used protein G-coated MPs and Gram-generic antibodies, Houser et al. [61] used magnetic particles coated with polydopamine to remove the bacteria for medical infections such as blood sepsis. Eight strains of bacteria were studied for this experiment, including *S. aureus*, *S. mutans*, *P. aeruginosa*, and three strains of *E. coli*. The capture efficiency was verified by measuring cell suspension turbidity (OD_600_) corresponding to bacteria not captured by the magnetic beads. The concentration of bacteria captured was determined by subtracting the concentration of non-captured bacteria from the concentration of bacteria incubated with the magnetic particles. Polydopamine functionalization was demonstrated to be highly effective [61] for MAC capture of Gram-positive cocci, Staphylococcus, and Streptococcus species, while a larger applicability for food control and water purification is also advocated.

Kang et al. [11] also used MPs to remove bacteria from blood samples. Magnetic particles coated with synthetic beta-2-glycoprotein I (sβ2GPI) peptides were employed to capture different bacterial strands. The entire process took less than one hour. Capture was effective for *E. coli* and *S. aureus* as well as for other bacterial strains like *K. pneumoniae*, *P. aeruginosa*, and *E. faecalis*. The collective detection limit for all the strains analyzed was under 4 CFU/mL [11].

Quintana-Sanchez et al. [62] used custom MPs functionalized with cationic carbosilane (CBS) dendrons and dendrimers for the recognition of *S. aureus* bacteria and *E. coli*. The bacteria were captured using the functionalized magnetic nanoparticles and then removed from the sample solution to be analyzed using SEM and FTIR. It was found that the magnetic particles with the higher density of cationic groups were the best ones for bacteria capture, with a limit of detection of 10^3^ cells/mL [62].

#### Analysis of Other Cells or Bioparticles

The use of MAC is not limited to pathogenic cells, and immunomagnetic capture was also applied for viruses. Tao et al. [37] developed magnetosome-like nanoparticles functionalized with methoxyl-polyethylene glycol2000-carboxyl and RBD-scFv antibodies for the detection of SARS-CoV-2. Despite their small sizes, these nanoparticles have a saturation magnetization of 90.6 emu/g compared with 98.5 emu/g of commercial magnetic particles. The magnetosome-like beads had a capture ability of 83 μg/mg [37].

Blood samples incubated with magnetic particles functionalized with the antibody anti-CD14 to detect CD14+ monocytes were analyzed using the FC technique in an article by Leuther et al. [40]. They could quantify the concentration of monocytes using the magnetic flow cytometer technique.

### 3.2. Circulating Tumor Cells

MAC is also a practical tool for the enrichment of circulating tumor cells (CTCs) that helps in their detection and analysis since conventional analysis methods like PCR are time-consuming and can take hours [60,63,64]. The separation of CTCs from blood has gained significant attention in recent years. However, capturing is difficult due to their low count (from 1–10 cells per 10 mL), heterogeneity, and potential for contamination. Numerous studies have been published regarding MAC since it offers significant advantages, such as high sensitivity and specificity, making it an attractive method for the isolation and analysis of CTCs.

In a recent work reported by Doswald et al. [65], magnetic particles coated with EpCAM antibodies, which label epithelial cell adhesion molecules, a transmembrane glycoprotein expressed on the surface of epithelial cells, were used to isolate CTCs and purify the blood-based samples. The magnetic particles did not interact with other blood components. Notably, in this assay, blood samples from healthy patients were spiked with HT-29 cells with a concentration of 5 × 10^5^ cells/mL, and in the real blood samples of patients with solid tumors, the CTCs were around 1 to 10 per mL. The samples, including blood from cancer patients, were analyzed using the flow cytometry technique.

In the work by Chang et al. [38], CTCs were captured by bimetallic magnetic gold nanoparticles coated with leukocyte membranes to form leukocyte membrane-camouflaged MPs functionalized with EpCam antibodies (CM-Fe_3_O_4_@Au-Ab). MAC was used to separate viable CTCs from impurities, as revealed by ~13× more target cells captured than leukocytes (that are in excess). The results of CM-Fe_3_O_4_@Au-Ab were encouraging, since cell sorting beads suffer from nonspecific aggregation and interaction with leukocytes that lead to inaccuracies and CTC detection limitation [38].

Wu et al. [66] present a study where magnetic particles coated with neutrophil membranes were used to separate CTCs from blood. With this type of immunomagnetic nanoparticles, they succeeded in isolating CTCs in 19 out of 20 blood samples from breast cancer patients, showing the role of magneto-immune separation in early noninvasive diagnosis of cancer [66]. Interestingly, this type of cell membrane coating of the MPs could effectively reduce nonspecific protein adsorption and improve the viability of isolated CTCs, as the cell membrane plays the role of a cushion, and thus can minimize the cell damage caused by interfacial collision.

Yu et al. [67] succeeded in creating floating immunomagnetic microspheres combining magnetic particles, poly(ethylene imine), hollow glass microspheres, biotinylated (poly(ethylene glycol))amine, and biotinylated anti-EpCAM antibodies for the detection of CTCs with a more cell-friendly technique that will not cause mechanical cellular damage. They tested these microspheres for four different cancer cell lines: human breast MCF-7, human lung A549, human liver HepG2, and human T-lymphocyte tumor Jurkat. Capture efficiency was as high as 93%, and the detection limit was 5 cells/mL [67].

Table 1 summarizes the principal components of the presented immunomagnetic capture, their target, and their detection limit. MAC’s principal advantage over the conventional plate counting method is time, considering that the process of immune separation can take less than an hour compared with around 12 h for plate counting.

Table 2 presents a summary of the advantages and disadvantages of these characterization methods, as well as the limit of detection found in the articles described.

#### Biomarkers

The topic of biomarker detection is not covered by this review. However, extracellular vesicles (EVs) have recently emerged as an attractive class of circulating biomarkers that share similarities with both cells and viruses. Availability of assays for EV detection in relation to MAC is therefore valuable. Termed integrated magnetic analysis of glycans in extracellular vesicles (iMAGE) [68], the technology utilizes EV glycan–lectin interactions in the solution phase to induce dual-selective aggregation of the MPs—that is specific to both EV biophysical characteristics and their glycan composition but unresponsive to free-floating glycoproteins—to cause magnetic signal changes in situ [68]. The resultant magnetic signals are quantified in real time through a built-in magnetoresistance sensor for direct analysis of EV glycans. When implemented on a miniaturized microfluidic platform, the iMAGE technology enabled rapid, wash-free, and multiplexed analysis of EV glycans in complex biofluids (<30 min, 1 μL of native samples). When applied to native clinical samples, the iMAGE technology not only revealed glycan signatures of cancer EVs against different biological backgrounds but also differentiated patient prognosis characteristics through direct EV glycan analysis.

**Table 2:** An overview of methods for characterization of magnetic immuno-clusters; TCID—tissue culture infective dose; CFU/mL—colony forming units.

Characterization Method	Type of Analysis	Advantages	Disadvantages	Limit of Detection	Ref.
Fluorescence microscopy assay	Mostly qualitative	Visualization of cluster formation, minimal sample preparation	Photobleaching, resolution limitation, quantitative challenges	-	[36]
Scanning electron microscopy (SEM)	Qualitative	Detailed 3D imaging of cluster formation, versatility	Cost Needs to be used in combination with other techniques for in-depth characterization	-	[29]
Electrical Impedance Spectroscopy (EIS)	Quantitative	Label-free detection, non-invasive	Limited information on cluster composition, complex data interpretation	1 ng/mL	[69]
Flow cytometry	Quantitative	Rapid and detailed size analysis	Struggle to measure large clusters, sample preparation accurately	2.5 ng/mL -	[21,65]
Surface-enhanced Raman spectroscopy (SERS)	Quantitative	Real-time monitoring, high spatial resolution	Instrumentation and expertise requirements; Analyzing mostly molecules on the surface of immunomagnetic clusters of those near the cluster surface	100 cells/mL 5.0 × 10^6^ TCID50/mL	[70,71]
Integrated magneto-affine selection, and magneto-phoretically assisted two-frequency electrical impedance assay	Quantitative	Label-free detection, non-invasive, information on cluster composition, automated data interpretation; Suitable for both large and small MPs; Concentration domain suitable for UTI assays. Suitable for phenotypic antimicrobial susceptibility testing	Novelty of the technique, with insufficient validation Not yet demonstrated for multiplexed formats	10^5^ CFU/mL	[22,32]

## 4. Open Challenges

An integrated analytical flow of immuno-clusters enables fast quantitation of target cells, expediting end-to-end results, and is suitable for complex samples. MAC allows improved capture efficiencies when combined with microfluidics through the enhancement of the encounter rate between the target and the affinity interface, and the improvement of binding kinetics. Binding the target directly alters the hydrodynamic volumes of the particles and leads to aggregation of magnetic particles into clusters with target concentration-dependent parameters. Cluster morphology and dynamics are dependent on the MP–(bio)particle relative ratios (of both dimensions and concentrations).

Cluster formation and control are demonstrated to be relevant when designing robust technologies suitable for portability and automated operation for fast and sensitive detection of target cells. The systems that use periodic actuation and lab-on-a-chip platforms to induce and record the oscillations of immuno-magnetically formed clusters of target cells have a fast analysis time and improved sensitivity. These are relevant for the development of screening tools for pathogen presence and cell viability evaluation in clinical and environmental samples.

### 4.1. Improvement of Magnetic Affinity Capture

The versatility and sensitivity of immunomagnetic capture depend significantly on the design and optimization of immunomagnetic elements, which must maintain high binding affinity, stability, and reproducibility under various experimental conditions [8]. A key factor influencing the success of MAC is the proper orientation of antibodies on the magnetic particles, as an improper one can impede target binding and reduce capture efficiency. Recent studies have focused on improving the antibody orientation, which enhances the reproducibility and sensitivity of MAC assays. These advancements hold great promise for further improving the performance of MAC in diagnostic and therapeutic settings [72,73].

Antibodies are the principal element used in the magnetic affinity assays. Antibodies are typically immobilized onto the solid surfaces of the capture beads and ideally should fulfil the three characteristics indicated in Gao [74], as follows:

(i) Firm conjugation on the matrices (e.g., covalent attachment);
(ii) Immobilized in a highly controllable and site-specific manner;
(iii) After functionalization, the final support should be demonstrated to be completely inert to avoid false positives (e.g., preventing unspecific interaction with either the detection antibodies or nonspecific clustering).

Coupling nucleophilic moieties (e.g., lysine residues) distributed on the antibody surface via imine bonds has been developed as a common strategy for immunosensing. This strategy results in randomly immobilized antibodies with only a fraction of the Fab binding site being available. Moreover, it does not enable orientation control, and the heterogeneous distribution of nucleophilic moieties hinders the availability of the antigen-binding fragment (Fab) region.

#### Immobilization Strategies Allowing Control of Antibody Orientation

For better results, it is essential to use immobilization strategies allowing control of antibody orientation [72,73]. The attachment of the Fc fragment in the Y-shaped commonly used antibodies in the immunoassays enables the orientation of the two Fab regions towards the antigens [72,73]. The correct orientation of immobilized antibodies is a key characteristic to capture large analytes with high sensitivity and selectivity, such as bacteria, coronavirus, and large proteins [74].

Therefore, one of the most common strategies for antibody orientation is coating magnetic beads with protein A or G. Due to their ability to selectively adsorb the Fc region of various types of antibodies, protein A (42 kDa) and protein G (58 kDa) provide an effective scaffold to regulate the orientation of full-length antibodies. As add-layers, the proteins facilitate the attachment of antibodies to magnetic beads to ensure the optimal antigen-binding orientation, do not interfere with the analyte-binding specificity after the adsorption process, and hence preserve antibody functionality and provide efficient and specific capture. Since no chemical modification is needed and the native binding properties are retained, these characteristics make them appealing candidates as intermediate layers for MAC sensors. Numerous scientific reports contain this type of antibody orientation strategy [72,73].

Focused comparison of actual antibody orientation capability is, however, scarcer. Laborie et al. [75] compared the free F(ab)2 fragments as a function of the MP add-layer. Protein G functionalized beads were compared with control (i.e., no add-layer), and tosyl- and streptavidin-functionalized ones. After immobilizing antibodies on the protein G beads, they found that around 75% of their F(ab)2 fragments remained free compared with 32% of the tosyl beads, 10% of the streptavidin beads, and only 5% of the control beads [75]. In another study [76], protein G and tosyl-functionalized beads were compared with non-functionalized carboxylic ones. All the beads were immobilized with NAB228 antibody, and then the ratios for F(ab’)2/Fc fragments released from NAB228 were determined. A higher amount of grafted NAB228 was found for tosyl and protein G activated beads, whereas for non-functionalized carboxylated ones, very little trace was found [76].

In an article by Oh et al. [77], protein A was used in combination with fusion tag protein glutathione-S-transferase (GST) for antibody orientation. The magnetic particles thus created were used for drug delivery for cancer therapy [77].

Another strategy for controlling antibody orientation is based on biotin–streptavidin linkage, since this interaction is one of the most powerful non-covalent attachments. In this strategy, the MB and the affinity compound should provide the complementary ligands, e.g., MPs functionalized with streptavidin and the affinity compound (capture antibodies) labelled with biotin. Alternatively, MPs can be biotinylated while streptavidin is attached to the antibody. In a study published by Carmody et al. [78], MPs coated with biotin were functionalized with a streptavidin-fused capsid protein (mSA-Hoc T4). This system was used to detect *E. coli* from water samples with a limit of detection of <10 CFU in 100 mL of water [78].

Instead of antibodies, MAC can incorporate aptamers for specifically targeting live cells. For instance, Cheng et al. [39] engineered two aptamers that were immobilized on streptavidin beads: rvCD71apt (reversible CD71 aptamer) and rvCD8apt (reversible CD8 aptamer) for detecting activated CD4+ T cells and resting CD8+ cells with high capture efficiency and specificity.

Other strategies for high-affinity functionalization with control of the orientation of the ligand include covalent immobilization of proteins with a histidine tag on particles functionalized with nitrilotriacetic acid. In Castro-Hinojosa et al. [79], magnetic particles were functionalized with ready-to-use nitrilotriacetic acid-divalent metal cation (NTA-M^2+^) complex and polyethylene glycol (PEG) molecules. They demonstrated the oriented immobilization of a cadherin fragment engineered with a hexahistidine tag (6His-tag) onto the MPs. This method is valuable since it could be extended to any protein containing a histidine tail, such as antibodies or enzymes [74,79]. Indeed, numerous recent studies have used this type of orientation for the magnetic particle surface [69,80,81].

According to the recent literature [82,83], orienting the ligands during MP functionalization improves the capture efficiency, and this control, together with procedures addressing the non-specific adsorption [84], warrants improvements of the overall sensitivity of the assay. However, there is no general procedure, and each assay development has to undergo specific design and validation.

### 4.2. Issues in System Development and Validation

#### 4.2.1. MP Quality

The size of the MP plays an important role in the effectiveness of MAC protocols and the sensitivity of the assay is largely influenced by the MP capturing capability, i.e., by the quality and freshness of the affinity-modified MPs: the differences in both the detection time and detection limit, while not always significant [60], can be associated to a reduced amount of antibody conjugated on the surface of smaller (e.g., 180 nm MBs [60]) when compared with larger (1 μm) MBs. Smaller-sized particles can penetrate impurities and minimize the nonspecific binding [37]; thus, their use was suggested as helpful in lowering the risk of false negative tests. Nevertheless, while the structure of clusters formed when using customized magnetic nanoclusters (CMNs) [22] is more specific to the captured microorganisms and there is a higher impact of the concentration of the captured bacteria in the overall CMN suggesting improved sensitivity, the small CMNs form rather easily (within a few days), microscopic compact clusters with inherently reduced capture capacity and altered dispersibility.

A time dependent decline in the effectiveness of the assay is evident even for large (1 μm) particles, the MPs’ affinity to the partner microbial cells gradually decreases, affecting the assay quality (with >75%, after 2 weeks since the preparation of the modified MP batches), especially in the high microbial concentration range.

There is a considerable array of commercial kits that provide ease of functionalization and effectiveness in tailoring MP-based protocols for immunomagnetic separation. However, as presented in the article by Arona et al. [85], where four different kits were compared for detecting Cryptosporidium from relatively simpler water samples, the effectiveness of the procedure can be highly variable. The magnetic particles from the kits were functionalized with various types of antibodies, like immunoglobulin IgG and IgM antibodies. Recovery of the bacteria was found between 75 and 93% for different samples using flow cytometry and immunofluorescence analysis [85].

Importantly, as demonstrated for nanoparticles coated with dendrimers [62], there is an inherent limit in the reusability of functionalized magnetic particles. Quintana-Sanchez et al. [62] reused MPs after each capture procedure in order to investigate the influence of CBS density on MP reusability. The capture decreased after each repetition as a function of CBS group density, with the MPs with the higher density of CBS groups suitable to be reused for a maximum of three times, and two times for the rest, when MPs were reused without a washing step. Removal of the bacteria by washing the MPs with a mix of ethanol and water and sonicating them for 5 min worsened the results and denatured the bacteria [62].

Eluting the bacteria from the magnetic particles, while maintaining their viability, has lately become a subject of interest in the scientific world since free, live bacteria can be used for subsequent bioanalysis, can be beneficial for characterization methods, and can reduce the general cost of analysis, enabling the reuse of magnetic particles. Numerous studies and protocols reported EDTA (Ethylenediaminetetraacetic acid) or high salt concentrations as an elution agent [86,87,88,89,90]. However, during our preliminary studies with micron-sized particles (unpublished data), we found that such protocols suggested an elution solution with EDTA and either dithiothreitol (DTT) or mercaptoethanol inefficient for bacteria detachment, even when using magnetic particles with disulfide bonds.

#### 4.2.2. Capture Efficiency in MAC

The capture efficiency (CE) is typically quantified as the percentage of the total number of cells retained on affinity-modified magnetic particles versus a known (spiked) concentration of the test culture, according to the following equation

CE% = (1 − C_sup_/C_cells_) × 100

where C_sup_ is the concentration of the cells that were not immuno-magnetically separated (remaining in the sample), and C_cells_ is the concentration of cells in the reference sample.

**Figure 6:**
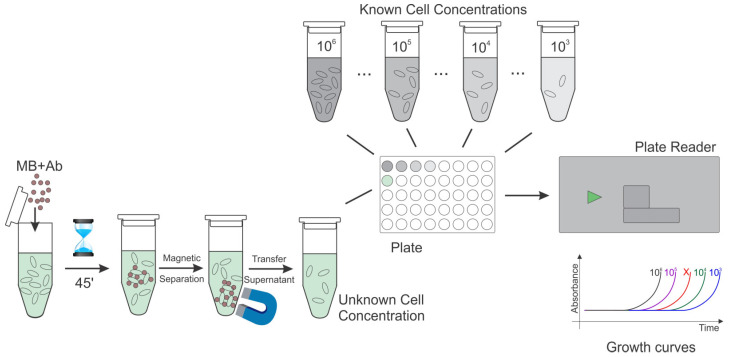
Schematic of the capture efficiency assay based on growth curves. Figure adapted from ref. [27]. Open access under Creative Commons CC-BY license.

These amounts are typically evaluated by plate count according to the scheme in Figure 6.

**Figure 7:**
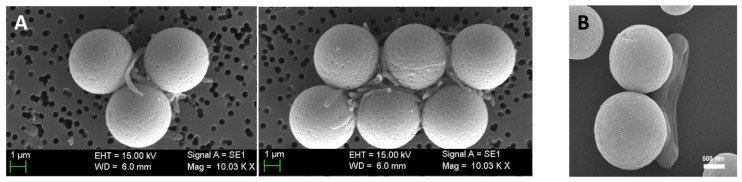
(**A**) Evaluation with SEM of the immunomagnetic capture of *Legionella pneumophila* cells at a concentration of 10^7^ CFU mL^−1^ attached to MBs. Figure reproduced from ref. [28]. Open access under Creative Commons CC-BY license (**B**): SEM images of *E. coli* O157:H7 captured by immunomagnetic beads. Scale bar 500 nm. Figure reproduced from ref. [29]. Open access under Creative Commons CC-BY license.

To determine the capture rate, a high-throughput assay can be implemented based on 96-well plate absorbance-derived growth curves of reference cultures. To this end, the MBs functionalized with affinity compounds are incubated with reference samples of various concentrations (used as the controls), undergo magnetic separation, and growth curves determined based on the absorbance changes of the cells in the supernatant are evaluated. The procedure is both time-consuming and imprecise for low target cell concentrations and reveals different growth dynamics for cells in the supernatant and when affinity-bound on the MPs (unpublished results). Moreover, affinity separation and clustering are dependent on time and experimental conditions. The CE in MAC procedures is dependent on the quality of the affinity-modified MP synthesis, the duration of the incubation, the dimension and number (i.e., MP–target cell ratio) of carrier beads used in the assay, and on the live/dead cell status [27]. The quality of the immunomagnetic beads is tailored by the type, concentration, and recognition potential of the affinity compound used in the functionalization of the immunomagnetic particles. Accordingly, CEs are extremely variable. For instance, Cheng et al. [39] reported capturing the cells with a purity between 85 and 93% [39]. The format used involved streptavidin beads that were immobilized with two types of aptamers for detecting activated CD_4_^+^ T cells and resting CD_8_^+^ cells. Kang et al. [11] used magnetic particles coated with synthetic beta-2-glycoprotein I (sβ2GPI) peptides to capture different bacterial strands from PBS and blood. Capture efficiency was around 96% for *E. coli* and 92% for *S. aureus*. Efficiency was tested as well for other bacterial strains like *K. pneumoniae*, *P. aeruginosa*, and *E. faecalis*, and all strains were successfully captured. Doswald et al. [65] reported that CTCs were isolated in a proportion of just over 68% using magnetic particles coated with EpCAM antibodies. By comparing SEM images (Figure 7) of samples before and after immuno-magnetic capture, one can loosely estimate the degree of target enrichment, information that is relevant when assessing the effectiveness of the capture method, yet the quantitative evaluation of capture efficiencies or cellular status is rarely demonstrated. Arona et al. [85] used flow cytometry and immunofluorescence analysis to evaluate quantitatively the capture efficiency of four different commercial kits for detecting *Cryptosporidium* from water samples. The magnetic particles from the kits were functionalized with various types of antibodies, like immunoglobulin IgG and IgM antibodies, and the recovery of the bacteria was found to be between 75 and 93%.

A thorough analysis [27] comparing carboxyl (CMB) and tosyl (TMB) Dynabeads towards affinity capture optimization revealed that the best capture efficiency (>75%) was achieved with TMB and live cells.

#### 4.2.3. Magnetic Affinity Capture Effect on Cell Viability and Dynamics

The fact that cell dynamics are different when attached to surfaces is a general paradigm in cell biology and medicine. MAC adds even more complexity to this issue, up to a point, especially when rare cells are targeted, and the cell-friendliness of MAC is considered a significant experimental challenge. For instance, aware of the possible mechanical cellular damage, Yu et al. [67] proposed creating floating immunomagnetic microspheres combining magnetic particles, hollow glass microspheres, and biotinylated anti-EpCAM antibodies for the detection of CTCs with a more cell-friendly technique.

MAC can induce cellular stress as revealed in SEM images, even for the rather robust bacterial cells. SEM is often used for characterization of immunomagnetic cluster formation, even though it is not suitable for routine verifications or field applications. SEM is handy for examining the morphology, size, surface properties, and distribution of the magnetic immuno-clusters formed during immunomagnetic capture [28,29] (Figure 7) and can help visualize the interaction between the beads and the target cells.

SEM can provide detailed images of how the magnetic beads interact with the surface of the microorganisms, shedding light on potential improvements in capture techniques for isolating cells or pathogens from complex biological samples and hints regarding the non-invasiveness of the capture. If bacteria are deformed from their original shape upon MP capture, this is a clear sign of stress, damage, or external pressure [61]. In Park et al. [29], SEM images are used to confirm cluster formation and immunomagnetic separation of *E. coli* with anti-*E. coli* O157 coupled magnetic beads (Figure 7A) with evident perturbed cell structure. Mesas Gomez et al. [28] evaluated the performance of direct immunomagnetic separation of bacteria retained on various filtering materials. SEM images highlight the high efficiency of the tosyl-functionalized magnetic particles to pull out the Legionella pneumophila from solid materials and reveal highly deformed cells. Moreover, when attempting to confirm capture of bacteria with affinity-functionalized (nano) MPs by visualizing the SEM image (Figure 7B), Houser et al. [61] noticed that *S. aureus* bacteria have bare patches without adherent beads and that the bacteria were deformed from their original spherical shape. The different cell dynamics when attached to affinity-modified MPs and undergoing MAC, as well as the concerns regarding capture efficiency and reproducibility, are important issues in method applicability and validation.

The potential of hybrid methods in advancing the field is addressed in the following section.

#### 4.2.4. Development and Validation of Hybrid Methods

- Hybrid electro-optic assays of MAC

Cluster formation and control are demonstrated as relevant when designing sensitive technologies suitable for portability and automated operation for fast and sensitive detection of target cells. The systems that use periodic actuation and lab-on-a-chip platforms to induce and record the oscillations of immuno-magnetically formed clusters of target cells have fast analysis times and improved sensitivity.

Indeed, a hybrid magnetically assisted electrical assay [22,32] provides high capture efficiency, analytic results within 30 min directly from unprocessed samples (buffer and synthetic urine), and high sensitivity in distinguishing live and dead cells in dynamic exposures. In principle, the system allows analysis of microbial susceptibility or resistance to selected antimicrobials, e.g., antibiotics/antifungal drugs, including pore-forming (membrane-disrupting) peptides [91,92], being a suitable support in the fight against antimicrobial resistance spread. Further testing and validation are, however, prerequisites, and MAC changes to the behavior of affinity-captured cells are possibly a confounding issue when testing cell susceptibility or resistance to certain treatments. This problem is further augmented due to the scarcity of quantitative assays and the lack of relevant reference data.

While EIS analysis is performed simultaneously at two AC frequencies [22] to detect and quantitate (derive the concentration) of magnetically tagged target cells, as well as their viability per se, or upon exposure to an antimicrobial compound, system validation will require evaluation with a wider frequency domain and possibly an integration of optical evaluation channel. The availability of electrically activated quantitative phase imaging platforms enabling high-resolution impedance mapping [93] is worth mentioning.

- Hybrid Surface-Enhanced Raman Spectroscopy (SERS);

Surface-enhanced Raman spectroscopy (SERS) is a highly sensitive technique that is valuable in measuring immunomagnetic clusters. SERS is based on enhancing Raman scattering through the interaction of light with molecules adsorbed on a metal nanostructure (usually gold, silver, or other plasmonic materials). This enhancement allows for the detection of very low concentrations of target analytes. Raman analysis, a non-destructive vibrational spectroscopy, is used for substance identification by analyzing molecular structures, composition, and environment of immunomagnetic clusters. Raman scattering molecules are photostable and enable the development of various SERS-based methods for detecting pathogens in complex sample matrices and in magnetic clusters [70,71].

In an article by Chattopadhyay [70], SERS was used to measure the concentration of the pathogen *S. typhimurium* in the range of 10^1^–10^7^ cells mL^−1^ with a limit of detection of 100 cells mL^−1^ by analyzing immunomagnetic clusters of magnetic particles with bacteria. The magnetic core of nanoparticles facilitated easy separation of target bacteria from the milieu of non-specific molecules. Gold nanoparticles (GNPs) modified with antibodies (CSA-1-Ab) and external Raman reporter molecules (RRMs) were used as signal probes. Capture and signal probes sandwich the target bacteria upon their addition. Under optimal conditions, the SERS intensities of MBA and DSNB at 1588 and 1336 cm^−1^, respectively, were used to measure the concentration of the pathogen in the range of 10^1^–10^7^ cells mL^−1^. The limit of detection (LOD) for two different reporter molecules was measured as 100 cells mL^−1^ and 10 cells mL^−1^, respectively. Moreover, appreciable recovery (82–114%) was recorded for the sensing method for different spiked food products. Thus, the developed magnetically assisted SERS immunosensor is sensitive, specific, and has strong potential to be used for detecting contamination in food samples in field conditions. The Raman signal intensity logarithm was directly proportional to the concentration of bacteria from 10–10^7^ cells mL^−1^. The test with SERS also showed the specificity of the magnetic particles for the *S. typhimurium* bacteria. The particles were incubated with 10^7^ cells mL^−1^ concentrations of *E. coli*, *P. aeruginosa*, *S. aureus*, *S. sonnie*, and *S. typhimurium*. The gold magnetic particles used provide imperceptible signal intensity at 1336 cm^−1^ for all other bacteria except *S. typhimurium*, even at high concentrations. This is due to the firm and specific binding between the capture antibody that tags MPs and the target bacteria, while the binding of the antibody with non-target bacteria is weak [70].

In another study by Wang et al. [71], research was conducted to verify if the H5N1 influenza virus can be detected from different samples using SERS. The results demonstrated a consistent Raman intensity at 1053 cm^−1^ across three independent H5N1 influenza virus samples, each tested in triplicate. The H5N1 influenza virus was diluted 10-fold, ranging from 5.0 × 10^6^ to 5.0 × 10^−7^ TCID50 mL^−1^ (TCID–tissue culture infective dose). The detection limit was 5.0 × 10^−6^ TCID50 mL^−1^ in the Raman spectra. The Raman peak height decreased as the H5N1 influenza virus concentration dropped, and the calibration demonstrated a linear relationship between the virus concentration and the Raman peak height at 1053 cm^−1^ [71].

**Figure 8:**
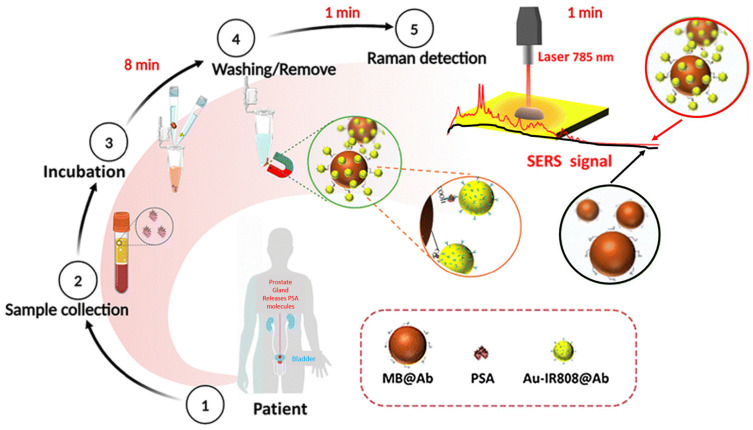
The analytic assay based on PSA antibody-coupled SERS nanotags and PSA antibody-coupled carboxyl magnetic beads for the detection of PSA antigen in serum. Figure adapted from ref. [94].

One key advantage of SERS is its high sensitivity, allowing for the detection of molecular interactions and structural features of clusters at very low concentrations. SERS enhances the Raman scattering signal by using metal nanoparticles, which amplify the signal from the captured target, making it particularly effective for detecting small clusters or rare biomolecules. The disadvantages of SERS are the need for precise control over the preparation and uniformity of the metal nanostructures, as variability in the plasmonic enhancement can affect the reliability and reproducibility of results, and the requirement of specialized equipment and expertise for data interpretation, which can limit its accessibility and applicability in some settings. A recent work [94] shows SERS detection on functionalized MPs. PSA antibody-coupled SERS nanotags and PSA antibody-coupled carboxyl magnetic beads were used (Figure 8) for the detection of PSA antigen in serum.

With the advent of multifunctional core-shell magnetic microspheres as a novel SERS-activity labels [95] and sensitive SERS assays in capillaries of electromagnetophoretic force induced molecular bond stretches [96], it appears convincing that powerful hybrid SERS methods of MAC cells will reach their full analytic potential.

## 5. Conclusions

Magnetic affinity capture enables efficient separation, enrichment, and purification of various target cells, thereby enhancing downstream analyses and facilitating high-throughput screening. With ongoing research and technological innovation, techniques for control and assessment of magnetic affinity particle clusters for live cell analyses offer more efficient, precise, and accessible solutions across a range of medical fields and thus hold the potential to significantly impact healthcare. Its utility spans various fields, including clinical diagnostics, cancer research, immunology, and environmental monitoring. This flexibility is enabled by the use of magnetic beads with various functionalities and affinity ligands.

Key aspects for improving magnetic affinity capture concern optimizing the orientation of the ligands on the magnetic beads as well as control of nonspecific binding, magnetic field, and assay characteristics. These enhance binding specificity and affinity, reduce steric hindrance, and minimize background noise, all of which contribute to improved capture efficiency and more reliable results.

Assessment and control of cluster formation are demonstrated to be relevant when designing sensitive technologies suitable for portability and automated operation for fast and sensitive detection of target cells. The systems that use periodic actuation and lab-on-a-chip platforms to induce and record the oscillations of magnetically affine clusters formed with target cells directly from unprocessed samples have high capture efficiency, fast analysis time, and improved sensitivity in distinguishing live and dead cells in dynamic exposures. New application fields concern the unmet needs for faster antibiotic susceptibility testing that is currently based on slow and resource-consuming bacterial pre-enrichment steps and lengthy growth-related assays. Novel hybrid assays supported by the integration of MAC in lab-on-a-chip procedures will warrant the achievement of new and improved test platforms for these types of microbiology analyses. Furthermore, by enabling real-time tracking of binding events and cluster dynamics, these hybrid assays appear ideal for cancer diagnostics or pathogen detection applications.

With careful control and validation, the perspectives for immunomagnetic clusters formation and capture are vast, with advancements expected to drive progress in diagnostics, personalized medicine, nanomedicine, and therapeutic applications.

## Figures and Tables

**Table 1:** An overview of immunomagnetic capture targets, components, and limits of detection; CTC—circulating tumor cells.

MP	Antibody	Target	Analysis Method	Limit of Detection	Ref.
Dynabeads Carboxylic Acid	*S. cerevisiae*-specific antibody	*S. cerevisiae*	Custom (magneto-phoretic assisted electrical impedance assay)	10^5^ cells/mL	[22]
Multivariate Metal-Organic Frameworks	- aptamer	*S. cerevisiae*	Fluorescence detection	10 cells/mL	[60]
Dynabeads Carboxylic Acid	Anti-*E. coli* O157:H7, anti-Gram-positive, anti-Gram-negative	*E. coli*	Fluorescence detection	10^2^ CFU/mL	[17]
Streptavidin Magnetic Beads	Anti-*E. coli* O157:H7	*E. coli*	Fluorescence spectrophotometer	0.35 CFU/mL	[56]
Carboxyl Magnetic Beads	Anti-EtpA IgG	*E. coli*	Fluorescence detection	Not specified	[54]
Coated with polydopamine	-	*S. aureus*, *S. epidermidis*, *S. mutans*, *N. perflava*, *P. aeruginosa* and 3 strains of *E. coli*	Optical density measurement	10 CFU/mL	[61]
Carboxyl Magnetic Beads	Anti-OmpF	*Y. enterocolitica*	PCR	64 CFU/mL	[15]
SiO_2_ Beads	Egg yolk antibody	*S. aureus*	Bioluminescence detector	3 CFU/mL	[58]
Fe_3_O_4_ Beads	EpCAM	CTCs	Microfluidic device	1–100 cells/mL	[65]
Fe_3_O_4_ Beads	Neutrophil membrane	CTCs	(immuno) fluorescence detection	Not specified	[66]
Dynabeads Streptavidin	Anti-human CD45 (AF488-anti-CD45) and biotinylated anti-human EpCAM	CTCs	Optical density measurement	5 cells/mL	[67]

## Data Availability

No new data were created or analyzed in this study.
